# Screening for Gastric and Small Intestinal Mucosal Injury with Magnetically Controlled Capsule Endoscopy in Asymptomatic Patients Taking Enteric-Coated Aspirin

**DOI:** 10.1155/2018/2524698

**Published:** 2018-11-15

**Authors:** Xue Chen, Feng Gao, Jie Zhang

**Affiliations:** Digestive Department, Beijing Anzhen Hospital, Capital Medical University, Beijing 100029, China

## Abstract

**Objective:**

To investigate gastric and small intestinal mucosal injury in asymptomatic patients taking enteric-coated aspirin using magnetically controlled capsule endoscopy.

**Methods:**

Patients taking enteric-coated aspirin (aspirin group) and healthy controls (control group) were recruited from Beijing Anzhen Hospital, Capital Medical University, between September 2017 and May 2018, and undertook magnetically controlled capsule endoscopy.

**Results:**

Twenty-six subjects were recruited to the aspirin group and twenty-six to the control group; the median Gastrointestinal Symptom Rating Scale scores were 3.50 and 3.00 (*P* = 0.200), the median gastric Lanza scores were 2.50 and 1.00 (*P* < 0.001), the small intestinal Lanza scores were 1.00 and 0.00 (*P* < 0.001), the gastric controlled examination times were 50.0 and 51.0 min (*P* = 0.171), the small intestinal transit times were 240.0 and 238.0 min (*P* = 0.654), and the capsule excretion times were 24.0 and 24.0 hours (*P* = 0.956), respectively.

**Conclusions:**

Rates of gastric and small intestinal mucosal injuries were significantly higher in patients without obvious gastrointestinal symptoms taking enteric-coated aspirin compared to healthy controls. Magnetically controlled capsule endoscopy constitutes a safe, real-time screening modality for gastric and small intestinal mucosal injury in patients taking enteric-coated aspirin.

## 1. Introduction

Aspirin is both a primary and secondary preventive drug for patients with cardiovascular diseases [1]. Aspirin inhibits platelet activation and thrombogenesis. However, it also injures the gastrointestinal mucosa through local and systemic actions [2, 3]. With the introduction of capsule endoscopy (CE) and double-balloon enteroscopy, reports on aspirin-related small intestinal mucosal injury are increasing year by year [4–6]. Gastroscopy is the most commonly used screening method for mucosal injury in the upper digestive tract. But, being an invasive modality, it can only be used to observe the upper digestive tract (esophagus, stomach, and duodenum). On the other hand, traditional CE can only screen the small intestinal mucosa [7]. Therefore, there is a lack of clinical methods to simultaneously screen for gastric and small intestinal mucosal injury in patients taking enteric-coated aspirin. Magnetically controlled capsule endoscopy (MCCE) is a new technique that can be used to screen for gastric mucosal lesions [8–10]. The aim of this study was to screen for gastric and intestinal mucosal injury using MCCE in asymptomatic patients taking enteric-coated aspirin.

## 2. Materials and Methods

### 2.1. Ethics

All research and data analysis methods were approved by the local ethics board of Beijing Anzhen Hospital, Capital Medical University.

### 2.2. Subject Selection

Healthy controls and patients without obvious gastrointestinal symptoms taking enteric-coated aspirin, aged 18–70 years, were enrolled from Beijing Anzhen Hospital, Capital Medical University, from September 2017 to May 2018.

Inclusion criteria included taking and not taking enteric-coated aspirin for more than 3 months without obvious gastrointestinal symptoms (abdominal pain, abdominal distension, diarrhoea, acid regurgitation, or heartburn), for the aspirin and healthy control groups, respectively.

Exclusion criteria included ① known or suspected digestive tract obstruction, fistula, or stenosis; ② history of gastrointestinal surgery; ③ cardiac pacemakers or metal implants; ④ pregnancy; ⑤ mental illness; ⑥ a positive C13 breath test; ⑦severe heart, lung, liver, or renal dysfunction; and ⑧ history of using other kinds of NSAIDs, gastric mucosa protective drugs, and proton pump inhibitor within 3 months before the procedure.

### 2.3. MCCE

The MCCE system was produced by Shanghai Ankon Medical Technologies Co. Ltd. (Shanghai, China) and Ankon Technologies Co. Ltd. (Wuhan, China) and included an endoscopic capsule, a capsule locator, a guidance magnet robot, check suit with data recorder, and a computer workstation running ESNavi software for real-time monitoring and controlling. The size of the capsule was 27.0 mm × 11.8 mm, the viewing angle was 140°, the image resolution was 480 × 480 ppi, and the frame rate was 1–2 frames/s (manually adjusted). There is a permanent magnet in the capsule that can be guided manually by the magnet robot. Data recording and downloading procedures were similar to those used for other traditional capsules.

### 2.4. Preparation for MCCE

The subjects fasted from 7 o'clock the night before the examination; they consumed two boxes of polyethylene glycol electrolytes powder (IV; Staidson Beijing Biopharmaceutical Co. Ltd.) with 1.5 l of clear water the night before the examination and again on the morning of the examination for cleansing of the small intestine. Gastric preparation included ① consuming simethicone power, a defoaming drug, to improve the intragastric view (10 g with 100 ml clear water 40 minutes before swallowing the capsule; Sichuan Jewelland Pharmaceutical Co. Ltd.); ② drinking 300 ml clear water 30 minutes before swallowing the capsule; ③ drinking 500 ml clear water before swallowing the capsule, activating the capsule, and swallowing the capsule; and ④ drinking 500 to 1000 ml clear water during the examination as required to keep the gastric cavity filled. All communication devices and ferromagnetic items, including watches, mobile phones, bank cards, glasses, metal ornaments, etc., should be removed from the subjects during the examination.

### 2.5. MCCE Control Protocol

Each subject lay down on the console. When the capsule reached the stomach, the operator could control the position of the capsule to ensure visibility of the gastric fundus, cardiac regions, gastric body, gastric angle, gastric antrum, pylorus, and duodenal bulb. Once the capsule had entered the duodenum, the subject could leave the console and return home wearing the check suit. The subject could eat 4 hours after the capsule had entered the duodenum and remove the check when it ran out of power. Following the examination, the subject was instructed to pay attention to every bowel movement and confirm whether the capsule had been excreted; magnetic resonance measurements were not possible until excretion of the capsule had been confirmed. If no capsule excretion was observed within 14 days, the subject was instructed to contact the operator and confirm the position of capsule by using the capsule locator or abdominal plain film. After the subjects had returned the check suit, the data from the check suit were exported to the computer workstation, and the images were observed and analysed by ESNavi software. All images were reviewed independently by two experienced doctors.

### 2.6. Data Collection

All subjects were assessed using the Gastrointestinal Symptom Rating Scale (GSRS) [11, 12]. The GSRS covers 15 gastrointestinal symptoms, each classified into four severity categories (score of 0–3).

The mucosal injury Lanza scores were as follows [13]: 0, no visible lesion; 1, mucosal erythema only; 2, 1–2 erosions; 3, several (3–10) erosions; and 4, large number (>10) of erosions or ulcers.

The gastric controlled examination time was the time of the capsule entering the duodenum minus the time of the capsule entering the stomach. The small intestinal transit time was the time of the capsule entering the cecum minus the time of it entering the duodenum. The capsule excretion time was the time of excretion of the capsule minus the capsule swallowing time.

### 2.7. Study Groups

The groups in this study comprised of patients taking enteric-coated aspirin (aspirin group) and healthy controls (control group).

### 2.8. Statistical Analyses

Categorical data were compared using the chi-square test and are presented as numbers. Continuous, normally distributed data were compared using independent sample *t*-tests and are presented as means ± standard deviation. Continuous, nonnormally distributed data were compared using the Mann-Whitney *U* test and are presented as medians (interquartile range (IQR)). Spearman correlation analysis was used to determine correlations between variables. A *P* value < 0.05 was considered statistically significant. All statistical analyses were performed using SPSS software (ver. 22.0; IBM Corp., Armonk, NY, USA).

### 2.9. Sample Size Calculation

The main index of the research was to evaluate the aspirin-related small intestine mucous injury. We prestudied 10 cases in each group, and the small intestinal Lanza scores were 1.2 ± 1.0 in the aspirin group and 0.2 ± 0.4 in the control group. PASS 11 software was used to calculate the sample size required. And each group needed at least 14 cases. We did 26 cases in each group that were able to fully meet the need to detect the difference.

## 3. Results

**Figure 1:**
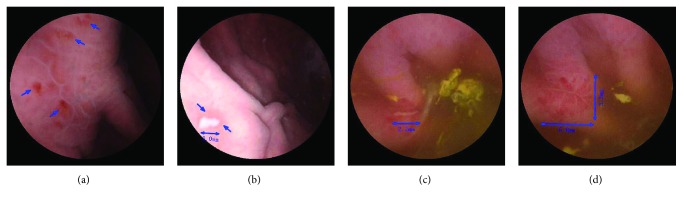
Magnetically controlled capsule endoscopic pictures of aspirin-related gastric mucosal injury. The blue arrows indicate injuries. (a) Gastric fundus erosion; (b) gastric fundus ulcer; (c) gastric antrum ulcer; (d) gastric antrum ulcer.

**Figure 2:**
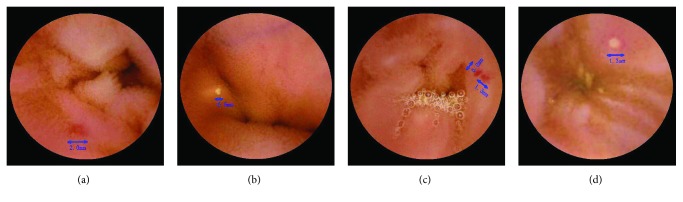
Magnetically controlled capsule endoscopic pictures of aspirin-related small intestinal mucosal injury. The blue arrows indicate injuries. (a) Jejunal erythema; (b) jejunal ulcer; (c) ileal erythema; (d) ileal ulcer.

Twenty-six subjects were included in the aspirin group, and twenty-six in the control group. The two groups showed no significant difference in age or gender (Table 1). The results of the aspirin and control groups were as follows: GSRS scores, 3.50 (7.00) and 3.00 (5.25) (*P* = 0.200); gastric Lanza score, 2.50 (2.15) and 1.00 (1.00) (*P* < 0.001); small intestinal Lanza score, 1.00 (0.25) and 0.00 (0.00) (*P* < 0.001); gastric controlled examination time, 50.0 (6.0) min and 51.0 (10.0) min (*P* = 0.171); small intestinal transit time, 240.0 (96.0) min and 238.0 (94.0) min (*P* = 0.654); and capsule excretion time, 24.0 (16.0) hours and 24.0 (10.0) hours (*P* = 0.956). In total, 84.6% (22/26) of patients taking enteric-coated aspirin suffered both gastric and small intestinal injuries, and gastric and intestinal mucosal injury were significantly associated (Spearman correlation coefficient, 0.662, *P* < 0.001). Magnetically controlled capsule endoscopic pictures of enteric-coated aspirin-related gastric and small intestinal mucosal injury are presented in Figures 1 and 2.

The capsule excretion rate was 100%. Most subjects excreted capsules within 24–48 hours. The longest excretion times were 120 hours (one subject in the control group) and 152 hours (one subject in the aspirin group). There were 49 cases (94.2%) showing complete tolerance to MCCE and 3 cases showing mild discomfort (5.8%). The mild discomfort was mainly related to cleansing of the small intestine, drinking simethicone, and drinking too much clear water. The main symptoms were nausea and upper abdominal discomfort, which resolved spontaneously within 24 hours.

## 4. Discussion

Aspirin injures the digestive tract mucosa through local and systemic actions, resulting in ulcer formation and bleeding and, in severe cases, potentially death. The incidence of gastroduodenal mucosal lesions has been reported as 48.4–63.1%, versus 10.7–31.7% for gastroduodenal ulcers and 57.6% for small intestinal mucosal lesions [2, 5]. The mortality rate of nonsteroidal anti-inflammatory drug- (NSAID-) related peptic ulcers is about 20–25 per million cases, of which one-third are attributable to low-dose aspirin (LDA) [14]. In middle-aged and elderly populations, LDA often leads to dyspepsia, epigastric pain, acid reflux, heartburn, and other symptoms, which reduces compliance; patients may stop taking aspirin, in turn increasing the risk of adverse cardiovascular events [15, 16].

There are multiple mechanisms of aspirin-induced gastrointestinal mucosal injury, which can be summarised as follows: ① direct injury to gastric mucosal epithelial cells; ② inhibition of the activity of cyclooxygenase-1, reducing mucosal flow and mucus and bicarbonate secretion, impairing platelet aggregation, interfering with prostaglandin synthesis, and inhibiting repair of the gastrointestinal mucosa; ③ inhibition of the activity of cyclooxygenase-2, reducing angiogenesis and increasing leukocyte adherence, resulting in gastrointestinal mucosal capillary stenosis or even occlusion, and decreasing gastrointestinal mucosal blood flow; ④ the antiplatelet effect of aspirin via inhibition of the aggregation of platelets on the surface of erosions and ulcers; and ⑤ participation of oxygen free radicals and self-digestion of pepsin in aspirin-related gastrointestinal mucosal injuries [2, 3]. Aspirin can injure the gastric and small intestinal mucosa at the same time; however, there is a lack of clinical methods allowing simultaneous screening for gastric and small intestinal mucosal injury.

The advantage of gastroscopy is that it can accurately identify the location of lesions and extract samples for biopsy. However, due the uncomfortable and invasive nature of the examination, many patients are reluctant to undergo nonanesthetic gastroscopy. Anesthetic gastroscopy is not widely applied in China because of the need for an anesthetist and breathing machine, as well as the risk of anesthesia-related adverse events and the high cost. CE was first introduced in 2000 and typically applied for examination of the small intestine, because it cannot be controlled autonomously in viewing the gastric cavity [17, 18]. Recent studies [8–10, 19] have shown that MCCE can reliably detect gastric lesions with comparable accuracy to gastroscopy. MCCE can screen the mucosa of the esophagus, stomach, and entire small intestine in one examination, which is not possible using traditional CE and gastroscopy. MCCE has already been widely used for screening and diagnosing digestive diseases.

Taking enteric-coated aspirin by the patient, mostly having cardiocerebrovascular disease or combining cardiopulmonary disease, is a relative contraindication to undertake gastroscopy under light or conscious sedation. In China, the anesthesiologists usually do not agree with light or conscious sedation for gastroscopy at the outpatient department for patients over 70 years of age with cardiocerebrovascular diseases. Moreover, light or conscious sedation for gastroscopy can only screen the mucosa of the esophagus, stomach, and proximal duodenum, while MCCE can screen the mucosa of the esophagus, stomach, and entire small intestine in one examination, with comparable image clarity. And light or conscious sedation for gastroscopy also requires the cooperation of an anesthesiologist, and anesthetic drugs have certain risks, such as respiratory inhibition, allergies, and blood pressure drop. MCCE can be performed without stopping enteric-coated aspirin. Therefore, MCCE is a good choice for patients taking enteric-coated aspirin with cardiocerebrovascular disease.

Considering MCCE's advantages of increased accuracy, reduced pain and discomfort, noninvasiveness, and the lack of a requirement to stop taking aspirin before the examination, we believe that MCCE is particularly suited to screening for gastric and small intestinal mucosal injury in patients taking enteric-coated aspirin. Our results showed that patients without obvious gastrointestinal symptoms taking enteric-coated aspirin still had significantly higher rates of gastric and small intestinal mucosal injury than healthy controls, with 84.6% patients in the aspirin group suffering from gastric and small intestinal injuries at the same time. Gastric and intestinal mucosal injuries were significantly associated. MCCE was found to be safe in patients taking enteric-coated aspirin, with any adverse events occurring during the study mainly being related to the preparation stage.

The cost of MCCE is higher than that of gastroscopy, so we should consider its cost-benefit issue. The subjects of our study were patients taking enteric-coated aspirin for primary and secondary prevention of cardiocerebrovascular diseases. Once fatal gastrointestinal bleeding occurs in these patients, the results of failing to detect and prevent bleeding in time are sometimes irreparable. Our results showed that in patients taking enteric-coated aspirin without gastrointestinal symptoms, 84.6% of them had small intestinal mucosal injuries. MCCE could not only screen the upper gastrointestinal tract but also screen the entire small intestine. MCCE could help to detect small intestinal mucous injury, diverticulitis, vascular malformation, and tumor, and patients with these diseases are at the risk of potentially fatal gastrointestinal bleeding. Proton pump inhibitor (PPI) is commonly used to prevent and treat enteric-coated aspirin-induced upper gastrointestinal injury, while studies [20–22] reported that PPI can exacerbate the small intestinal mucosal injury. For patients taking enteric-coated aspirin for primary and secondary prevention of cardiocerebrovascular disease, it is not enough to just pay attention to the upper gastrointestinal diseases. Many people also have small intestinal lesions, such as diverticulitis, vascular malformation, and tumor. Patients with these lesions are at higher risk of gastrointestinal bleeding, even fatal gastrointestinal bleeding after taking enteric-coated aspirin. And MCCE can detect these patients in advance. Therefore, in patients having gastroduodenal lesions with or without small intestinal lesions, the clinical outcome is different, and the detection of small intestinal lesions by MCCE is beneficial.

Indeed, the procedure of MCCE took a little longer time. But, our subjects were patients taking enteric-coated aspirin, and they did need this test without stopping enteric-coated aspirin. And 94.2% of the subjects showed complete tolerance to the procedure of MCCE.

Our study had some limitations. First, all subjects were drawn from a single center, which might have introduced a selection bias. Nevertheless, we are the first to use MCCE to observe gastric and small intestinal mucosal injuries in patients without obvious gastrointestinal symptoms taking enteric-coated aspirin.

The rates of gastric and small intestinal mucosal injury in patients without obvious gastrointestinal symptoms taking enteric-coated aspirin were significantly higher than those in the healthy controls. MCCE constitutes a safe, real-time screening modality for the examination of gastric and small intestinal mucosal injury, which furthermore does not prevent the patient from continuing their aspirin prescription.

## Figures and Tables

**Table 1:** Demographic and clinical characteristics of the patients taking enteric-coated aspirin and healthy controls.

Item	Aspirin group	Control group	*P* value (chi-squared test or independent samples *t*-test or Mann-Whitney *U* test)
	*n* = 26	*n* = 26	
Age (mean ± SD, years)	57.53 ± 9.07	55.46 ± 6.69	*t* = 0.939 *P* = 0.352
Male/female (*n*)	16/10	14/12	*χ* ^2^ = 0.315 *P* = 0.575
GSRS	3.50 (7.00)	3.0 (5.25)	*Z* = −1.281 *P* = 0.200
Gastric Lanza score (median (IQR))	2.50 (2.15)	1.00 (1.00)	*Z* = −4.442 *P* < 0.001
Gastric ulcer (%)	0 (0)	3 (11.5)	*χ* ^2^ = 3.184 *P* = 0.235
Gastric controlled examination time (median (IQR), min)	50.0 (6.0)	51.0 (10.0)	*Z* = −1.370 *P* = 0.171
Small intestinal Lanza score (median (IQR))	1.00 (0.25)	0.00 (0.00)	*Z* = −4.761 *P* < 0.001
Small intestinal ulcer	0 (0)	2 (7.7)	*χ* ^2^ = 2.080 *P* = 0.490
Small intestinal transit time (median (IQR), min)	240.0 (96.0)	238.0 (94.0)	*Z* = −0.449 *P* = 0.654
Capsule excretion time (median (IQR), hour)	24.0 (16.0)	24.0 (10.0)	*Z* = −0.055 *P* = 0.956

Aspirin: patients taking enteric-coated aspirin; control: healthy controls; SD: standard deviation; GSRS: Gastrointestinal Symptom Rating Scale; IQR: interquartile range.

## Data Availability

The data used to support the findings of this study are available from the corresponding author upon request.
